# Role of Genetic Ancestry in 1,002 Brazilian Colorectal Cancer Patients From Barretos Cancer Hospital

**DOI:** 10.3389/fonc.2020.00145

**Published:** 2020-03-04

**Authors:** Ronilson Oliveira Durães, Gustavo Noriz Berardinelli, Allini Mafra da Costa, Cristovam Scapulatempo-Neto, Rui Pereira, Marco Antônio Oliveira, Denise Peixoto Guimarães, Rui Manuel Reis

**Affiliations:** ^1^Molecular Oncology Research Centre, Barretos Cancer Hospital, Barretos, Brazil; ^2^Department of Medical Oncology, Barretos Cancer Hospital, Barretos, Brazil; ^3^Cancer Registry, Barretos Cancer Hospital, Barretos, Brazil; ^4^Department of Pathology, Barretos Cancer Hospital, Barretos, Brazil; ^5^IPATIMUP (Institute of Molecular Pathology and Immunology of the University of Porto), Porto, Portugal; ^6^i3S (Instituto de Investigação e Inovação em Saúde, Universidade Do Porto), Porto, Portugal; ^7^Nucleous of Epidemiology and Statistics, Barretos Cancer Hospital, Barretos, Brazil; ^8^Endoscopy Department, Barretos Cancer Hospital, Barretos, Brazil; ^9^Life and Health Sciences Research Institute (ICVS), Medical School, University of Minho, Braga, Portugal; ^10^ICVS/3B's-PT Government Associate Laboratory, Guimarães, Portugal

**Keywords:** colorectal cancer, genetic ancestry, cancer outcome, colorectal cancer survival, prognostic factors

## Abstract

**Background:** Colorectal cancer (CRC) is the third most frequent and the second deadliest cancer worldwide. The ethnic structure of the population has been gaining prominence as a cancer player. The purpose of this study was to determine the genetic ancestry of Brazilian CRC patients. Moreover, we intended to interrogate its impact on patients' clinicopathological features.

**Methods:** Retrospective observational cohort study with 1,002 patients with CRC admitted from 2000 to 2014 at Barretos Cancer Hospital. Following tumor DNA isolation, genetic ancestry was assessed using a specific panel of 46 ancestry informative markers. Survival rates were obtained by the Kaplan–Meier method, and the log-rank test was used to compare the survival curves. Multivariable Cox proportional regression models were used to estimate hazard ratios (HRs).

**Results:** We observed considerable admixture in the genetic composition, with the following average proportions: European 74.2%, African 12.7%, Asian 6.5%, and Amerindian 6.6%. The multivariate analysis for cancer-specific survival showed that clinical stage, lymphovascular invasion, and the presence of recurrence were associated with an increased relative risk of death from cancer (*p* < 0.05). High African proportion was associated with younger age at diagnosis, while high Amerindian proportion was associated with the mucinous histological subtype.

**Conclusions:** This represents the larger assessment of genetic ancestry in a population of Brazilian patients with CRC. Brazilian CRC patients exhibited similar clinicopathological features as described in Western countries.

**Impact:** Genetic ancestry components corroborated the significant admixture, and importantly, patients with high African proportion develop cancer at a younger age.

## Introduction

Colorectal cancer (CRC) represents more than 1,849,518 new cases, accounting for approximately 10.2% of all neoplasms worldwide (1). CRC is the third most common neoplasm in men and the second in women (1–4). CRC mortality is also high, with 880,792 deaths having been estimated for 2018, corresponding to 9.2% of the total, with higher rates (52%) being observed in less developed regions of the world (1). The incidence of CRC varies more than 10-fold worldwide (1). The highest detection rates are observed in Australia, New Zealand, and countries of Europe and North America, and the lowest are found in the countries of Africa, South America, and Asia (1, 2). In Brazil, according to the National Cancer Institute (INCA), an estimated 17,380 new cases of colon and rectum cancer in men and 18,980 in women are expected for 2018, which occupies the third position in men and the second among women (4).

Several reasons account for these discrepancies, including distinct risk factors. Age is the primary risk factor, yet many other factors contribute to CRC development including a previous history of colorectal neoplasia and/or adenomas or family history of CRC; a diet rich in red meat and saturated fats, fruits, and vegetables; obesity and sedentary lifestyle; smoking; diabetes mellitus; CRC-associated syndromes such as familial adenomatous polyposis, hereditary non-polyposis colorectal cancer (HNPCC or Lynch syndrome); and inflammatory diseases of the colon (2, 3, 5, 6). Besides the risk factors abovementioned, patient ethnicity has been reported as a risk and prognostic factor (7–12). The ethnic structure becomes more pressing in the Brazilian population due to its great admixture (13–17). Currently, genetic markers are available that determine, with more assertiveness than the self-declared form or based on physical traits, the ethnic structure of each individual (14, 18).

Despite the high incidence and mortality rate of CRC in Brazil, few studies have comprehensively described and characterized the main clinicopathological features of Brazilian patients with CRC (19, 20). Therefore, this study aimed to characterize the clinicopathological aspects of CRC patients, to determine their genetic ancestry, and to identify whether the genetic ancestry can influence patients' clinicopathological features and disease outcome.

## Methods

### Study Design and Data Source

We conducted a retrospective observational cohort study enrolling 1,002 patients with CRC admitted from 2000 to 2014 at Barretos Cancer Hospital, Barretos, São Paulo, Brazil. Of the total of 1,002 cases, 96.3% (965/1,002) were selected from the Department of Low-Digestive and 3.7% (37/1,002) were oncogenetic-based cases, being 1.8% (18/1,002) confirmed Lynch syndrome cases, 1.5% (15/1,002) confirmed familial adenomatous polyposis (FAP) syndrome cases, and 0.4% (4/1,002) of unclassified hereditary syndrome (21). Clinicopathological and treatment data of CRC patients were collected from patient medical records. The present study evaluated 21 variables. The seventh edition of the American Joint Committee on Cancer (AJCC) was used for tumor staging. The Institutional Ethics Committee approved the study (protocol number: 600/2012-CAAE: 02468812.30000.5437).

### Genetic Ancestry Determination

DNA samples were recovered from formalin-fixed paraffin-embedded (FFPE) tissue of tumor specimens obtained from surgical or endoscopic procedures. The DNA was isolated using the DNA Micro kit (Qiagen), according to the method previously established by our group (22).

The ancestry of the patients was determined using ancestry informative markers (AIMs) as previously reported (14, 23–25). Briefly, 46 small insertion–deletion (INDEL) polymorphisms were ascertained to maximize the divergence between four human major population groups: Amerindian (AME), European (EUR), African (AFR), and East Asian (ASN). These markers were selected due to their high allele frequency divergence between different ancestral or geographically distant populations, including more than 1,000 individuals from 40 reference populations from the Human Genome Diversity Project (HGDP)-Centre d/Etude du Polymorphisme (CEPH), plus individuals from Angola, Portugal, Taiwan, and indigenous Brazilian, which allowed to establish the ancestral proportions in high admixture individuals and populations, like the Brazilian one (14). Moreover, they were assembled in a simple multiplex reaction following a short amplicon strategy, adequate for challenging samples such as FFPE (15, 26, 27). The primer sequences and PCR conditions were according to Giolo et al. (14).

After DNA extraction, and multiplex PCR with 46 primers, the amplified products were further subjected to capillary electrophoresis and fragment analysis on an ABI 3500 Genetic Analyzer (Applied Biosystems) according to the manufacturer's instructions. These 46 INDELs are used mainly to estimate ancestry proportions in admixed populations and assess the structure of those populations. Two observers independently analyzed the electropherograms, and the genotypes were automatically assigned with GeneMapper Software v4.1 (Applied Biosystems).

The ancestry ratios were evaluated using the Structure Software v2.3.4 (23, 24, 28, 29), considering the four main population groups, AME, EUR, AFR, and ASN, as possible contributors to the current Brazilian genetic composition. Briefly, the data available for the HGDP-CEPH panel were used as a reference for the ancestral populations, and a supervised analysis was performed to estimate ancestry relationship proportions of the individuals involved in the study. The Structure software runs considering K = 4 consisted of 100,000 burning steps followed by 100,000 Markov Chain Monte Carlo iterations. The option “Use population information to test for migrants” was used with the admixture model, considering allele frequencies correlated, and updating allele frequencies using only individuals with POPFLAG = 1.

### Statistical Analyses

Patient and cancer characteristics were reported as frequencies (number and percentage). First, the continuous variables of genetic ancestry component were summarized as mean [standard deviation (SD)]. For the association of the genetic ancestry component (AFR, EUR, ASN, AME) by AIMs panel with patient and clinical characteristics, the chi-square test or Fisher's exact test was used. For this step, ancestry proportions were further categorically defined as low, intermediate, and high based on tercile distribution (Table 1 and Supplementary Table 1).

**Table 1:** Ancestry background categorization according to tercile based on percentage proportions for all four ethnic groups.

**Ancestry components**	**Low**	**Intermediate**	**High**
AFR	<0.030	0.030–0.120	>0.120
EUR	<0.710	0.710–0.870	>0.870
ASN	<0.030	0.030–0.050	>0.050
AME	<0.030	0.030–0.060	>0.060

The overall survival (OS) and the cancer-specific survival (CSS) rates were obtained using the Kaplan–Meier method. Survival rates were estimated in months. Survival was defined as the period from diagnosis to the date of death or the time at which information was last obtained. For the analysis, the event of interest was death by any cause for OS and death related to cancer for CSS. Cases that were alive were censored for OS, and cases that were alive or dead from other causes were censored for CSS. Such information was obtained through direct consultation to the death certificate or medical records. The follow-up median of our sample was 62.0 months. The log-rank test was used to compare survival curves, and results were considered significant when the *p* < 0.05.

Multiple confirmatory models were used to check whether genetic ancestry component (AFR, EUR, ASN, AME) by AIMs panel was related to the prognosis of CRC. Multivariable Cox proportional hazards regression models were used to estimate hazard ratios (HRs) and 95% confidence intervals (CIs) for the variables with *p* < 0.20 in univariate analyses and adjusted with treatment period and genetic ancestry components by AIMs panel. Fisher exact test was used for association analysis.

For tabulation and statistical analysis, the IBM® SPSS® Statistics 21.0 software for Windows (IBM Corporation, Route 100, Somers, NY 10589) was used. The level of statistical significance was set at 0.05 for all analyses.

## Results

### Clinicopathological Features

The present study included 1,002 cases, and the main clinicopathological features are summarized in Table 2. A detailed description of therapeutic regimens is shown in Supplementary Table 2. There were more men than women in the population (51.9%), most patients were between 50 and 75 years old at diagnosis (60.5%), and the majority lived in the South or Southeast regions of Brazil (82%). The distribution of the CRC patients according to the Brazilian state of origin is plotted in Supplementary Figure 1. The left colon was the leading primary tumor site, representing 46% of the cases, and adenocarcinoma was the main histological type, representing 93.5% of the cases. The cases were distributed in all stages, but clinical stages II and III were the most common, representing together 70.8% of the cases.

**Table 2:** Clinicopathological features of Brazilian colorectal cancer patients (*n* = 1,002).

**Variable**	**Categories**	***n***	**%**
Gender	Male	520	51.9
	Female	482	48.1
Age at diagnose	<50	290	28.9
(in years)	>= 50 to <75	606	60.5
	>= 75	106	10.6
Brazilian region of origin	South / Southeast	822	82.0
	Midwest	87	8.7
	North / Northeast	93	9.3
Primary tumor site	Right colon	250	25.0
	Left colon	466	46.5
	Rectum	285	28.5
	Missing data	1	
Clinical stage (AJCC)	0/I	125	12.5
	II	376	37.6
	III	332	33.2
	IV	167	16.7
	Missing data	2	
Histological type	Adenocarcinoma	937	93.5
	Mucinous	58	5.8
	Others	7	0.7
Histological grade	I / II	924	93.6
	III / Undifferentiated	63	6.4
	Missing data	15	
Lymphovascular invasion	No	621	67.4
	Yes	300	32.6
	Missing data	81	
Perineural invasion	No	731	85.6
	Yes	123	14.4
	Missing data	148	
Presence synchronous tumors	No	944	94.2
	Yes	58	5.8
Presence of recurrence	No	698	69.7
	Yes	304	30.3
Treatment period	2000–2009	184	18.4
	2010–2014	818	81.6
Surgery	No	40	4.0
	Yes	961	96.0
	Missing data	1	
Neoadjuvant chemotherapy	No	18	10.2
	Yes	159	89.8
	Not applicable	824	
	Missing data	1	
Adjuvant chemotherapy	No	543	55.1
	Yes	442	44.9
	Missing data		
Radiotherapy	No	793	79.1
	Yes, neoadjuvant	165	16.5
	Yes, adjuvant	21	2.1
	Yes, paliative	21	2.1
	Missing data	2	
Vital status	Alive without cancer	465	46.4
	Alive with cancer	48	4.8
	Death for cancer	422	42.1
	Death for others causes	67	6.7

### Genetic Ancestry

**Figure 1:**
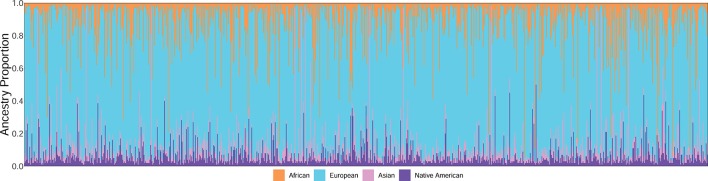
Individual ancestry estimates for the Brazilian colorectal cancer patients (*n* = 934).

**Figure 2:**
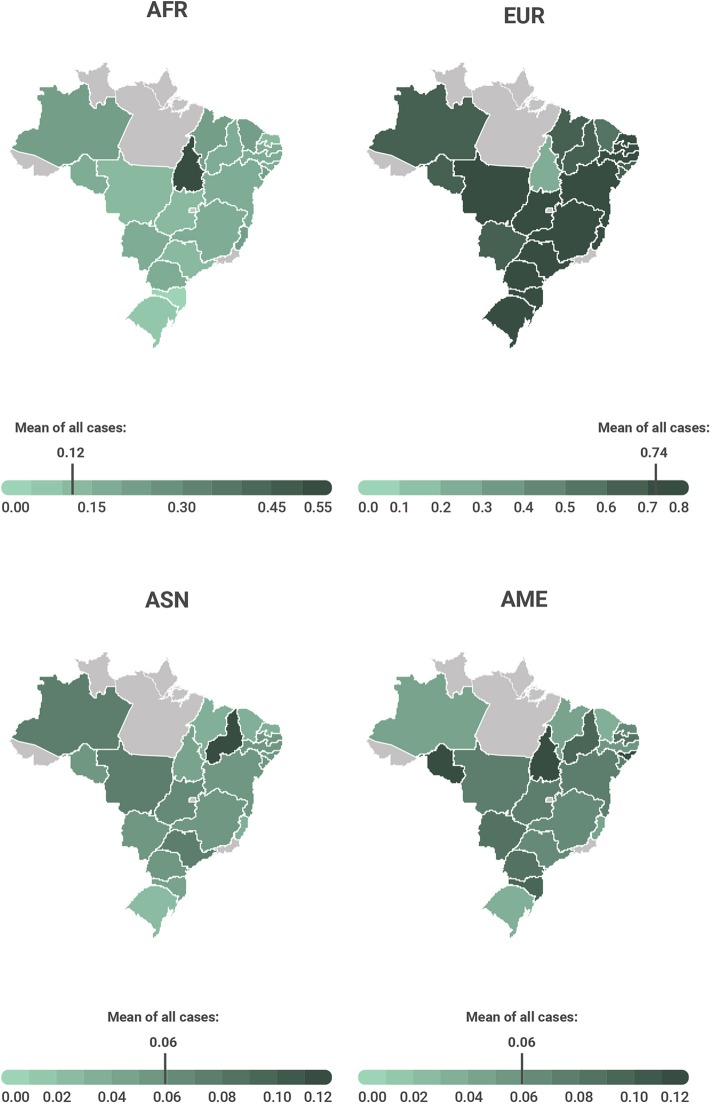
Average of each genetic ancestry component according to the Brazilian state of origin. AFR- African; EUR- European; ASN- Asian; AME- Amerindian (Native American).

The present study also aimed to evaluate the genetic ancestry of the patients, which was performed in 934/1,002 (93.2%) of the cases. In a small subset of cases (*n* = 68), the genetic ancestry could not be evaluated due to low quantity and poor-quality DNA. We observed a great admixture in genetic composition, with the following averages of ancestral proportions: AFR 12.7% (SD = 15.7%), EUR 74.2% (SD = 20.6%), ASN 6.5% (SD = 11.3%), and AME 6.6% (SD = 7.1%) (Figure 1). The average of each genetic ancestry component according to the Brazilian state of origin is plotted in Figure 2. The ancestry proportions were further categorically defined as low, intermediate, and high based on tercile distribution (Table 1 and Supplementary Table 1).

We further investigated the association of genetic ancestry with patients' clinicopathological characteristics (Table 3). We observed significant associations between the AFR component and younger age at diagnosis (*p* = 0.013), Brazilian region of origin (*p* < 0.001), and recurrence of the disease (*p* = 0.034). For the EUR component, we found significant associations with the region of origin (*p* < 0.001), adenocarcinoma (*p* = 0.023), higher histological grade (*p* = 0.040), and presence of synchronous tumors (*p* = 0.012). For the AME component, a significant association with the mucinous histological type (*p* = 0.033) was observed.

**Table 3:** Association between clinicopathological and genetic ancestry components by AIM-INDEL panel (*n* = 934).

**Variable/Categories**	**AFR**				**EUR**				**ASN**				**AME**			
	**Low *n* (%)**	**Int. *n* (%)**	**High *n* (%)**	***p*-value**	**Low *n* (%)**	**Int. *n* (%)**	**High *n* (%)**	***p*-value**	**Low *n* (%)**	**Int. *n* (%)**	**High *n* (%)**	***p-*value**	**Low *n* (%)**	**Int. *n* (%)**	**High *n* (%)**	***p*-value**
**GENDER**																
Male	189 (54.5)	153 (55.2)	149 (48.1)	0.149	155 (49.1)	174 (55.2)	162 (53.5)	0.277	234 (52.6)	114 (55.6)	143 (50.4)	0.517	212 (52.5)	123 (53.2)	156 (52.2)	0.969
Female	158 (45.5)	124 (44.8)	161 (51.9)		161 (50.9)	141 (44.8)	141 (46.5)		211 (47.4)	91 (44.4)	141 (49.6)		192 (47.5)	108 (46.8)	143 (47.8)	
**AGE AT DIAGNOSE (IN YEARS)**																
<50	74 (21.4)	87 (31.4)	96 (31.0)	**0.013**	99 (31.3)	82 (26.0)	76 (25.1)	0.453	115 (25.8)	59 (28.8)	83 (29.2)	0.528	118 (29.2)	59 (25.6)	80 (26.8)	0.701
≥50 to <75	225 (64.8)	164 (59.2)	185 (59.7)		185 (58.5)	198 (62.9)	191 (63.1)		275 (61.8)	129 (62.9)	170 (59.9)		240 (59.4)	150 (64.9)	184 (61.5)	
≥75	48 (13.8)	26 (9.4)	29 (9.3)		32 (10.2)	35 (11.1)	36 (11.8)		55 (12.4)	17 (8.3)	31 (10.9)		46 (11.4)	22 (9.5)	35 (11.7)	
**BRAZILIAN REGION OF ORIGIN**																
South/Southeast	308 (88.8)	226 (81.6)	232 (74.8)	**<0.001**	237 (75.0)	262 (83.2)	267 (88.1)	**<0.001**	369 (82.9)	163 (79.5)	234 (82.4)	0.160	346 (85.6)	187 (81.0)	233 (78.0)	0.052
Midwest	25 (7.2)	32 (11.6)	28 (9.1)		32 (10.1)	27 (8.6)	26 (8.5)		42 (9.5)	15 (7.3)	28 (9.9)		34 (8.5)	21 (9.0)	30 (10.0)	
North/Northeast	14 (4.0)	19 (6.8)	50 (16.1)		47 (14.9)	26 (8.3)	10 (3.3)		34 (7.6)	27 (13.2)	22 (7.7)		24 (5.9)	23 (10.0)	36 (12.0)	
**PRIMARY TUMOR SITE**																
Right colon	76 (22.0)	77 (27.8)	84 (27.1)	0.331	77 (24.4)	88 (27.9)	72 (23.8)	0.178	119 (26.7)	49 (24.0)	69 (24.3)	0.417	103 (25.6)	62 (26.8)	72 (24.1)	0.335
Left colon	171 (49.4)	125 (45.1)	133 (42.9)		136 (43.0)	140 (44.5)	153 (50.7)		211 (47.5)	95 (46.6)	123 (43.3)		197 (48.8)	102 (44.2)	130 (43.5)	
Rectum	99 (28.6)	75 (27.1)	93 (30.0)		103 (32.6)	87 (27.6)	77 (25.5)		115 (25.8)	60 (29.4)	92 (32.4)		103 (25.6)	67 (29.0)	97 (32.4)	
**CLINICAL STAGE (AJCC)**																
0/I	47 (13.6)	37 (13.4)	36 (11.6)	0.580	36 (11.4)	42 (13.4)	42 (13.8)	0.833	60 (13.5)	27 (13.2)	33 (11.7)	0.540	51 (12.7)	29 (12.6)	40 (13.4)	0.877
II	129 (37.3)	112 (40.6)	114 (36.8)		118 (37.3)	122 (38.9)	115 (38.1)		172 (38.7)	74 (36.1)	109 (38.5)		157 (39.0)	89 (38.5)	109 (36.6)	
III	108 (31.2)	92 (33.3)	106 (34.2)		114 (36.1)	97 (30.9)	95 (31.5)		150 (33.8)	62 (30.2)	94 (33.2)		136 (33.7)	70 (30.3)	100 (33.6)	
IV	62 (17.9)	35 (12.7)	54 (17.4)		48 (15.2)	53 (16.8)	50 (16.6)		62 (14.0)	42 (20.5)	47 (16.6)		59 (14.6)	43 (18.6)	49 (16.4)	
**HISTOLOGICAL TYPE**																
Adenocarcinoma	325 (93.6)	261 (94.2)	285 (91.9)	0.429	294 (93.0)	286 (90.8)	291 (96.0)	**0.023**	418 (93.9)	191 (93.2)	262 (92.3)	0.429	382 (94.6)	219 (94.8)	270 (90.3)	**0.033**
Mucinous	19 (5.5)	13 (4.7)	24 (7.8)		22 (7.0)	24 (7.6)	10 (3.3)		24 (5.4)	14 (6.8)	18 (6.3)		19 (4.7)	9 (3.9)	28 (9.4)	
Others	3 (0.9)	3 (1.1)	1 (0.3)		0 (0.0)	5 (1.6)	2 (0.7)		3 (0.7)	0 (0.0)	4 (1.4)		3 (0.7)	3 (1.3)	1 (0.3)	
**HISTOLOGICAL GRADE**																
I/II	317 (93.0)	259 (94.9)	285 (93.4)	0.612	298 (95.8)	282 (91.0)	281 (94.3)	**0.040**	404 (92.4)	193 (95.1)	264 (94.6)	0.332	374 (94.4)	214 (93.0)	273 (93.2)	0.713
III/Undifferentiated	24 (7.0)	14 (5.1)	20 (6.6)		13 (4.2)	28 (9.0)	17 (5.7)		33 (7.6)	10 (4.9)	15 (5.4)		22 (5.6)	16 (7.0)	20 (6.8)	
**LYMPHOVASCULAR INVASION**																
No	211 (64.7)	186 (71.0)	195 (69.9)	0.209	212 (73.1)	189 (64.5)	191 (67.3)	0.075	282 (67.8)	127 (69.0)	183 (68.5)	0.951	258 (68.4)	138 (64.8)	196 (70.8)	0.370
Yes	115 (35.3)	76 (29.0)	84 (30.1)		78 (26.9)	104 (35.5)	93 (32.7)		134 (32.2)	57 (31.0)	84 (31.5)		119 (31.6)	75 (35.2)	81 (29.2)	
**PERINEURAL INVASION**																
No	252 (84.8)	208 (84.6	230 (87.1)	0.657	235 (86.4)	234 (84.5)	221 (85.7)	0.812	327 (85.8)	152 (87.9)	211 (83.4)	0.425	299 (86.2)	171 (83.4)	220 (86.3)	0.617
Yes	45 (15.2)	38 (15.4)	34 (12.9)		37 (13.6)	43 (15.5)	37 (14.3)		54 (14.2)	21 (12.1)	42 (16.6)		48 (13.8)	34 (16.6)	35 (13.7)	
**PRESENCE SYNCHRONOUS TUMORS**																
No	332 (95.7)	260 (93.9)	285 (91.9)	0.135	288 (91.1)	305 (96.8)	284 (93.7)	**0.012**	417 (93.7)	196 (95.6)	264 (93.0)	0.469	381 (94.3)	211 (91.3)	285 (95.3)	0.149
Yes	15 (4.3)	17 (6.1)	25 (8.1)		28 (8.9)	10 (3.2)	19 (6.3)		28 (6.3)	9 (4.4)	20 (7.0)		23 (5.7)	20 (8.7)	14 (4.7)	
**PRESENCE OF RECURRENCE**																
No	238 (68.6)	188 (67.9)	237 (76.5)	**0.034**	232 (73.4)	217 (68.9)	214 (70.6)	0.450	311 (69.9)	144 (70.2)	208 (73.2)	0.602	286 (70.8)	170 (73.6)	207 (69.2)	0.544
Yes	109 (31.4)	89 (32.1)	73 (23.5)		84 (26.6)	98 (31.1)	89 (29.4)		134 (30.1)	61 (29.8)	76 (26.8)		118 (29.2)	61 (26.4)	92 (30.8)	

*Significant associations are indicated in bold*.

### Survival Analysis

An initial univariate analysis of survival was performed, including 1,002 individuals: 489 events occurred in OS, and 422 events occurred in CSS. The probability of patients living for more than 5 years was 58.2% for OS and 62.3% for CSS (Table 4). Several significant associations were observed between OS and CSS and patients' features, including gender, clinical stage, histological type, histological grade, lymphovascular invasion, perineural invasion, presence of recurrence, treatment period, neoadjuvant chemotherapy, adjuvant chemotherapy, and radiotherapy (Table 3 and Supplementary Figure 2). On the univariate survival analyses (OS and CSS), the genetic ancestry categorically defined as low, intermediate, and high based on terciles was not associated with CRC survival (Table 4).

**Table 4:** Kaplan-Meier estimates of overall survival and cancer-specific survival of colorectal cancer patients (*n* = 1,002).

**Variable**	**Categories**	**Cases**	**Global survival**			**Cancer-specific survival**		
			**Deaths**	**5 years**	***p*-value**	**Deaths**	**5 years**	***p*-value**
All cases		1,002	489	58.2		422	62.3	
Gender	Male	520	275	54.4	**0.004**	236	58.9	**0.011**
	Female	482	214	62.3		186	65.8	
Age at diagnose	<50	290	134	62.0	0.066	133	62.4	0.368
(in years)	≥50 to <75	606	299	57.0		257	61.4	
	≥75	106	56	55.0		32	67.7	
Brazilian region of origin	South / Southeast	822	405	57.9	0.514	352	61.7	0.609
	Midwest	87	37	65.3		32	69.8	
	North / Northeast	93	47	54.0		38	59.9	
Primary tumor site	Right colon	250	111	60.0	0.289	90	65.7	0.168
	Left colon	466	221	58.9		195	62.2	
	Rectum	285	156	55.6		136	59.6	
Clinical stage (AJCC)	0/I	125	29	86.4	**<0.001**	15	93.9	**<0.001**
	II	376	126	72.5		97	78.2	
	III	332	175	54.4		156	57.8	
	IV	167	157	13.6		154	13.9	
Histological type	Adenocarcinoma	937	458	58.5	**0.004**	395	62.6	**0.009**
	Mucinous	58	25	60.1		22	62.2	
	Others	7	6	14.3		5	17.1	
Histological grade	I/II	924	441	59.6	**0.001**	380	63.7	**0.002**
	III/Undifferentiated	63	41	37.4		35	41.1	
Lymphovascular	No	621	227	69.7	**<0.001**	180	74.9	**<0.001**
invasion	Yes	300	200	40.6		184	42.8	
Perineural invasion	No	731	301	64.9	**<0.001**	248	69.8	**<0.001**
	Yes	123	89	35.4		85	36.0	
Presence synchronous	No	944	463	58.2	0.967	403	62.1	0.487
tumors	Yes	58	26	58.6		19	65.3	
Presence of recurrence	No	698	245	67.8	**<0.001**	182	74.2	**<0.001**
	Yes	304	244	36.5		240	36.9	
Treatment period	2000–2009	184	142	49.5	**<0.001**	138	50.2	**<0.001**
	2010–2014	818	347	60.3		284	65.3	
Surgery	No	40	38	7.5	**<0.001**	37	7.7	**<0.001**
	Yes	961	450	60.4		385	64.6	
Neoadjuvant chemotherapy	No	18	14	38.9	**0.002**	10	46.7	**0.034**
	Yes	159	80	59.1		71	62.7	
Adjuvant chemotherapy	No	543	313	49.4	**<0.001**	260	54.6	**<0.001**
	Yes	442	169	69.1		157	71.0	
Radiotherapy	No	793	376	58.6	**<0.001**	322	62.8	**<0.001**
	Yes, neoadjuvant	165	85	58.7		73	63.0	
	Yes, adjuvant	21	7	81.0		7	81.0	
	Yes, paliative	21	20	19.0		20	19.0	
AFR	Low	347	171	58.0	0.403	148	62.2	0.308
	Intermediate	277	139	56.7		119	60.8	
	High	310	137	60.2		114	64.9	
EUR	Low	316	140	61.3	0.247	117	66.0	0.228
	Intermediate	315	160	55.0		136	59.1	
	High	303	147	58.8		128	63.0	
ASN	Low	445	215	57.6	0.833	184	62.1	0.819
	Intermediate	205	101	59.2		86	63.2	
	High	284	131	58.8		111	63.3	
AME	Low	404	190	58.9	0.859	167	63.0	0.949
	Intermediate	231	109	58.7		95	61.6	
	High	299	148	57.2		119	63.2	

*Significant associations are indicated in bold*.

The multivariate analysis for CSS adjusted by treatment period and genetic ancestry components showed that clinical stage, lymphovascular invasion, and the presence of recurrence were associated with an increased relative risk of death from cancer (p < 0.05), whereas adjuvant chemotherapy was associated with a lower risk of death (Table 5). These results are explained by the different therapeutic approaches used in distinct clinical stages (Supplementary Tables 3, 4).

**Table 5:** Multivariate analysis of cancer-specific survival associated with different clinicopathological characteristics and treatment of patients with colorectal cancer.

**Variables**	**Categories**	***Crude***			Adjust 1^*^		
		**RR**	**IC 95%**	***p***	**RR**	**IC 95%**	***p***
Gender	Male	1	–	–	1	–	–
	Female	**0.77**	**0.61–0.97**	**0.031**	**0.73**	**0.57–0.93**	**0.011**
Age at diagnose	<50	1	–	–	1	–	–
(in years)	≥50 to <75	1.16	0.90–1.50	0.240	1.22	0.93–1.61	0.140
	≥75	1.23	0.79–1.93	0.347	1.31	0.82–2.11	0.252
Clinical stage (AJCC)	0/I	1	–	–	1	–	–
	II	**2.68**	**1.40–5.14**	**0.003**	**3.22**	**1.58–6.58**	**0.001**
	III	**4.89**	**2.50–9.57**	**<0.001**	**6.16**	**2.96–12.83**	**<0.001**
	IV	**11.46**	**5.99–21.91**	**<0.001**	**14.01**	**6.87–28.58**	**<0.001**
Histological type	Adenocarcinoma	1	–	–	1	–	–
	Mucinous	0.58	0.33–1.04	0.070	0.53	0.28–1.00	0.051
	Others	1.13	0.33–3.82	0.840	1.04	0.30–3.61	0.947
Histological grade	I/II	1	–	–	1	–	–
	III/Undifferentiated	1.13	0.74–1.72	0.552	**1.05**	**0.67–1.65**	**0.817**
Lymphovascular	No	1	–	–	1	–	–
invasion	Yes	**1.55**	**1.17–2.04**	**0.002**	**1.53**	**1.15–2.04**	**0.003**
Perineural invasion	No	1	–	–	1	–	–
	Yes	1.17	0.88–1.56	0.264	1.12	0.83–1.51	0.454
Presence of recurrence	No	1	–	–	1	–	–
	Yes	**3.49**	**2.74–4.45**	**<0.001**	**3.38**	**2.63–4.35**	**<0.001**
Treatment period	2000–2009	1	–	–	1	–	
	2010–2014	0.98	0.75–1.28	0.898	0.92	0.69–1.22	0.572
Surgery	No	1	–	–	1	–	–
	Yes	0.56	0.07–4.50	0.587	0.64	0.07–5.22	0.679
Adjuvant chemotherapy	No	1	–	–	1	–	–
	Yes	**0.58**	**0.42–0.79**	**0.001**	**0.55**	**0.40–0.76**	**<0.001**
Radiotherapy	No	1	–	–	1	–	–
	Yes, neoadjuvant	1.02	0.74–1.39	0.902	1.08	0.78–1.49	0.637
	Yes, adjuvant	0.76	0.33–1.75	0.526	0.72	0.29–1.80	0.487
	Yes, paliative	1.93	0.90–4.10	0.088	1.94	0.90–4.16	0.089

* *Adjust 1: Genetic ancestry components by AIM-INDEL panel*.

*NA, Not available*.

*Significant associations are indicated in bold*.

## Discussion

CRC is one of the most common neoplasms in men and women worldwide (3, 30, 31). Although its incidence is declining in the US and other western countries (32); in others, including Brazil, we are still witnessing an increase in the number of cases, and it is a major public health problem. In this study, we intended to characterize the genetic ancestry of an extensive series of 1,002 CRC patients admitted at the Barretos Cancer Hospital. Knowing that the Brazilian population is ethnically one of the most heterogeneous in the world (14, 18), with an essential contribution from the main ethnicities that formed the background of our population, we also intended to correlate the ancestry components (EUR, AFR, ASN, and AME) measured genetically with the different clinical–pathological factors and its prognostic role.

There was a slight male predominance, with an incidence of 1.08. In all regions of the world, despite the similarities between genders, the rates were higher for males (vs. females, 1.3) in the American population (2, 33), as well as in Europe (1) and Asia (34). Others have a higher incidence among women in the colon (4, 35).

The main studies divide the samples into three age categories: below 50, between 50 and 75, and above 75 years old. The age of 50 years old is critical to differentiate between hereditary and sporadic CRC cases. This age limit has been used in the Amsterdam criteria (36, 37) and also to recommend screening colonoscopic examination for people at average risk for CRC (38, 39). Although it has been reported that 21 to 33% of patients are older than 75 years [Surveillance Epidemiologic and End Results (SEER)] (40), they may account for more than 40% and are underrepresented in the clinical studies. These clinical studies use in their inclusion criteria an age group of up to 75 years old as a limit to be treated (41–44), mainly due to comorbidities. Therefore, we adopted the upper limit range as those with 75 or more years old (45).

The mean age at diagnosis in our population was 57.7 years (SD = 13.8), below the American age of 68 years (31) and the European age of 72 years (45). The predominant age group in our population was between 50 and 75 years old (60.5%), similar to that in the SEER (31) data.

Our population had a high incidence of patients younger than 50 years old (28.9%), higher than the 20% reported in studies including North American populations (31) and Asian patients (3–14%) (46). This finding can be due to the inclusion criteria and to the potential presence of some hereditary cases in the present analysis. In the present study, patients with a known and genetically confirmed familial history of Lynch or APC represented <4% of cases (21); however, we cannot rule out the existence of hereditary cases in the cohort. Since 1992, the incidence in cases under 50 has increased by 1.5% per year (3, 45), especially from 20 to 34 years. According to the American College of Gastroenterology (39), colorectal cancer screening begins at age 50, except for those of African origin, where it is recommended to start at age 45 (47). Moreover, some studies even question to initiate at 40 years old (48). In concordance with these findings, we observed that Brazilian CRC patients depicting higher African proportion were associated with younger age of disease onset.

The importance of primary tumor location, being associated with distinct clinical–pathological features, as well as a differential prognostication has been widely discussed. For this, we performed the categorization of the cases included into the right colon, left colon, and rectum (49–53). In our population, 25% of the tumors were in the right colon. This percentage is within the average of other studies that ranged from 22.7 to 39% (49). However, in contrast, we did not find that laterality was associated with disease outcome.

Another critical variable is the TNM staging. In our study, the majority of cases were stage II (37.6%), followed by III (33.2%) and IV (16.7%). The percentage of stage IV at diagnosis is in agreement with several regions of the world (31, 45, 54).

Another goal of our study was to evaluate the main prognostic factors in our CRC patients. To this end, we estimated the OS and CSS and correlated with the different variables collected and selected in the multivariate analysis. The follow-up median of our sample was 62.0 months, very similar to the SEER that was 65.2 months (31). In the study of OS and CSS, we interrogated whether the variables selected in the multivariate analysis would be influenced by other variables such as the treatment period and the genetic ancestry components. Therefore, following adjustment of both variables, namely, treatment period (patients treated from 2000 to 2009 and from 2010 to 2014, where the introduction of the molecular target drugs, such as cetuximab, were included by the Department of Oncology of the Barretos Cancer Hospital), and ancestry, a multivariate analysis was performed.

The multivariate analysis for OS and CSS adjusted by genetic ancestry showed that the clinical stage, lymphovascular invasion, and recurrence of the disease were associated with an increased relative risk of death from cancer. In contrast, adjuvant chemotherapy was associated with a better outcome, as expected.

About 1/3 of our patients had lymphovascular invasion. The association of lymphovascular dissemination and adverse outcomes (55, 56) is well-described, besides being a known definer regarding therapeutics, especially in stage II (3).

The ancestry of the individuals assumes importance concerning its association with specific pathologies, immunological, and therapeutic responses, yet in the vast majority of studies, it is not evaluated (57, 58). Currently, with the availability of molecular tools for genetic studies, self-declaration and/or family origin can no longer be a proxy/authentication of the ancestral origin of an individual or population, especially in regions with a high degree of population admixture such as Brazil (57). However, in a large number of studies, skin color alone is used to assert ethnical origin of CRC patients (7–9, 12, 59). There is an extensive amount of studies, based on self-declaration, suggesting that black skin color patients have a higher incidence and lower survival of CRC (7, 12). However, it is unclear whether the ancestral component alone would influence survival or whether there are other confounding factors, such as less reference to screening methods (60), presence of a higher number of comorbidities diagnosis (7), lack of access to treatment services (61), or low educational/economic level, which can justify this fact (11, 60).

There are several ways to analyze the genetic composition of a particular population, and the selection criteria of genetic markers may diverge between studies, originating different values of the ethnic groups (62) for the Brazilian population. In our study, we analyzed four major ethnic compositions (EUR, AFR, ASN, and AME) using AIMs according to previous studies (13, 14, 18, 63). The present study was retrospective, dependent on the collection of information in medical records, and there was no mention of self-declaration of skin color. Therefore, our data collection instrument did not contemplate this aspect. If we had this information, we could have carried out a cross-referencing of information between the genotype and the phenotype to try to evaluate the fidelity of the latter.

When ancestry was measured genetically, we did not evaluate the data individually, but rather the four ancestor components that form the demographic base of Brazil. Our study did not intend to assess the causal relationship between genetic ancestry and CRC cancer as already done in other studies (64), but rather to correlate them with the various clinical–pathological characteristics of the patients.

As expected, the predominant ancestral component was the EUR one, with an average of 74% followed by the AFR with 13%, and by the ASN and AME with 7%, agreeing with previous studies of the Brazilian population (13, 63, 65). In agreement with other studies (62), a predominance of the European ancestral component in the Southeast and South regions was observed (63). Some differences in our study were observed, for example, the African component concentrated more strongly in the north region, unlike other studies based on the mitochondrial DNA (mtDNA) and not on autosomal AIM-*INDELs*, where this happened more in the Northeast Brazilian region (13, 17, 62, 63). The contribution of Asian ancestry in the northeast region of 5% is very close to that of the regions known to be colonized by Asians, but this may perhaps be explained first by the small sample size representing this region in our study and/or the proximity of the gene pool between Amerindians and eastern Asians considering the modern history of these human groups (14). The high SDs identified in our study show how miscegenated our Brazilian population is.

When we evaluated the individual components separately, we found that the European ancestral component was significantly associated with the absence of synchronous tumors. The African component was associated with younger patients, in agreement with other international studies (7, 8, 10). The Amerindian component predominated in the Northern region which correlates with other studies and is corroborated by the IBGE (Brazilian Institute of Geography and Statistics) self-declaration assessment (13, 18, 63, 66). Interestingly, we observed an association of the Amerindian component with mucinous histological type.

In our study, there was no correlation between the different ancestry proportions and patient survival. However, some North American studies reported an association of African ancestry based on self-declaration with tumors located in the right colon (67) and that these would be associated with more aggressive behavior histopathology, which would lead to worse survival (7, 11, 12).

Finally, despite the exciting and important findings, this study harbors some limitations, such as the retrospective nature of the study, based on the analysis of medical records, which often do not have complete and accurate information. The extent of the AIM panel could also be arguably higher. Nonetheless, the employed AIM indel set harbors a sufficient number of markers sparsely distributed throughout the genome and is simply analyzed in a multiplexed short-amplicon strategy, which are desirable characteristics considering the challenging nature of the source tumor samples included in our study. Despite the large number of patients and their diverse geographic origin, it does not represent all Brazilian states and the fully ethnical diversity of the Brazilian population, so further studies are warranted to extend our findings.

## Conclusion

This pioneering work determined the genetic ancestry profile of more than 1,000 Brazilian patients diagnosed with CRC from a single oncology reference center. We described the main clinicopathological features of the population and observed that patients with a high African proportion develop cancer at a younger age. The present study can contribute to drawing a nationwide portrait of Brazilian CRC patient and may help in the design of management strategies for these patients.

## Data Availability Statement

The raw data supporting the conclusions of this manuscript will be made available by the authors, without undue reservation, to any qualified researcher.

## Ethics Statement

The studies involving human participants were reviewed and approved by Barretos Cancer Hospital Ethics Committee. Written informed consent was not provided because this was a retrospective study.

## Author Contributions

RD participated in study design, collection of the data, analyzed the data, and prepared the manuscript. GB participated in the collection of the data, carried out the ancestry experiments, and analyzed the data. AC participated in the collection of the data, analyzed the data, and preparation of the manuscript. CS-N participated in the collection of the data, re-evaluation of the diagnosis, and participated in the ancestry experiments. RP participated in the ancestry experiments and analyzed the data. MO participated in the statistical analysis and analysis of the results. DG participated in study design and analysis of the results. RR participated in study design, coordination, analysis of the results, and prepared the manuscript. All authors read and approved the final manuscript.

### Conflict of Interest

The authors declare that the research was conducted in the absence of any commercial or financial relationships that could be construed as a potential conflict of interest.

## Abbreviations

AIMs: ancestry informative markers
AFR: African
AME: Amerindian
ASN: Eastern Asian
CCR: colorectal cancer
CI: confidence interval
DNA: deoxyribonucleic acid
EUR: European
FFPE: formalin-fixed paraffin-embedded
HNPCC: hereditary non-polyposis colorectal cancer
HR: hazard ratio
INCA: National Cancer Institute
OS: overall survival
CSS: cancer-specific survival
D: standard deviation.
