# Supplementary figures and images for: Role of Genetic Ancestry in 1,002 Brazilian Colorectal Cancer Patients From Barretos Cancer Hospital
Source: Front Oncol. 2020 Mar 4;10:145. doi: 10.3389/fonc.2020.00145 (PMC7065467; doi:10.3389/fonc.2020.00145)

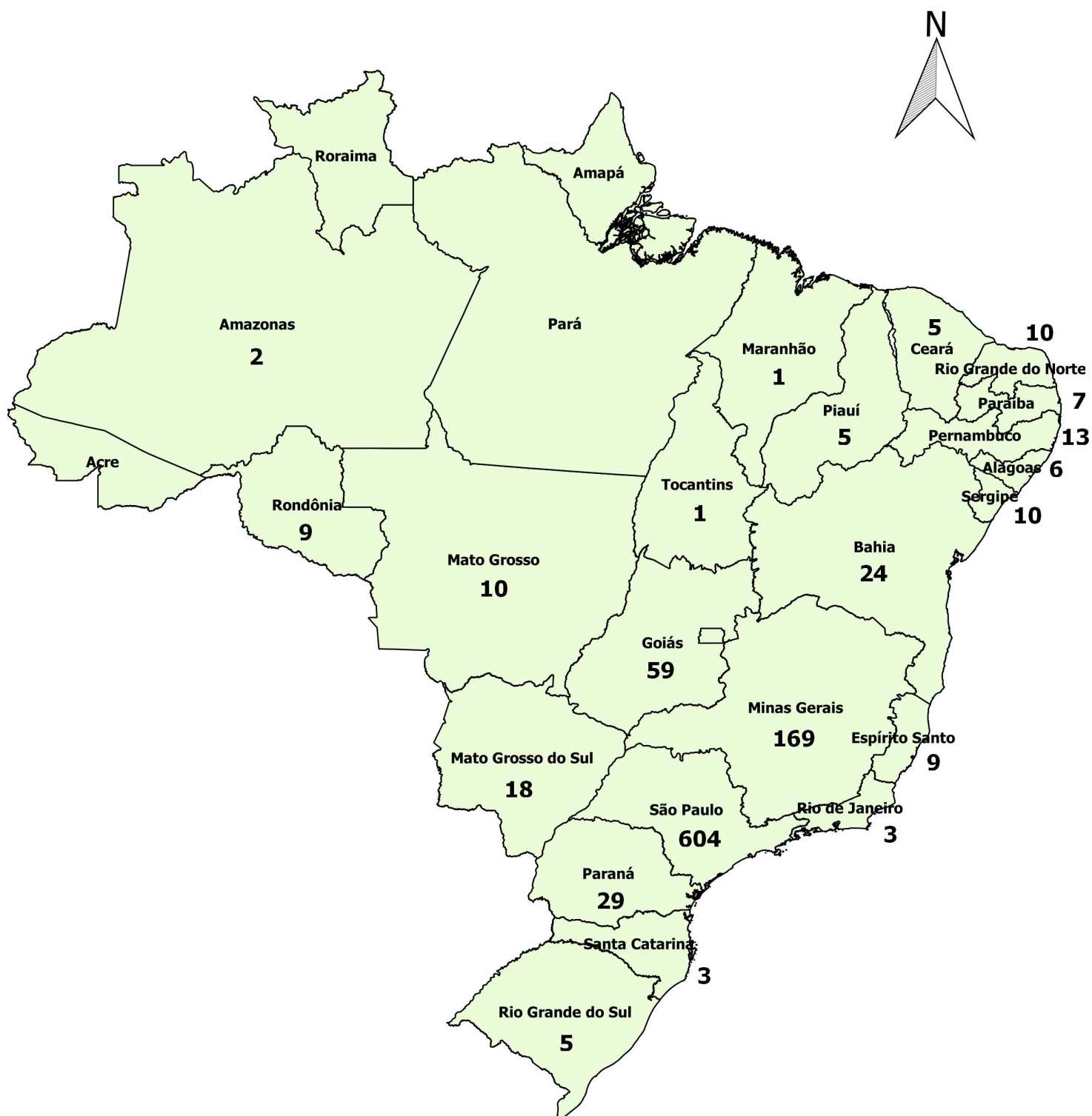

## Legend

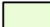 Brazilian states with the sampling frequency (n=1,002)

250 0 250 500 750 1000 km

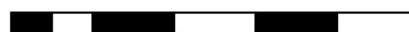

Supplement: Supplementary file 1 [file Image_1.pdf]
