# Supplementary material for: Role of Genetic Ancestry in 1,002 Brazilian Colorectal Cancer Patients From Barretos Cancer Hospital
Source: Front Oncol. 2020 Mar 4;10:145. doi: 10.3389/fonc.2020.00145 (PMC7065467; doi:10.3389/fonc.2020.00145)
Supplement: Supplementary file 2 [file Image_2.pdf]

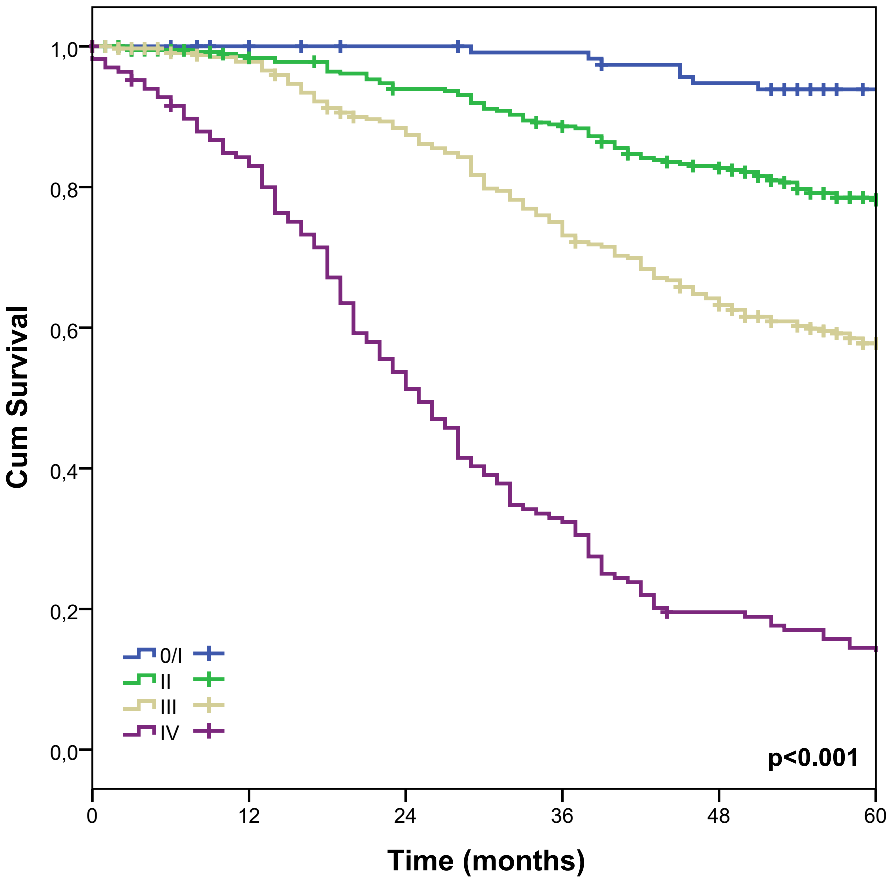

Supplementary Figure 2 - Kaplan-Meier curves for overall survival of colorectal cancer patients according to clinical stages
Survival time is presented in months; p values are related to Log- rank test results.
