# Supplementary material for: Role of Genetic Ancestry in 1,002 Brazilian Colorectal Cancer Patients From Barretos Cancer Hospital
Source: Front Oncol. 2020 Mar 4;10:145. doi: 10.3389/fonc.2020.00145 (PMC7065467; doi:10.3389/fonc.2020.00145)
Supplement: Supplementary file 3 [file Data_Sheet_1.pdf]

Supplementary Material

Supplementary Table 1. Genetic ancestry components by AIM-INDEL panel (n= 934).

Variable	Categories	n	%
AFR	Low (<0.030)	347	37.2
	Intermediate (0.030 - 0.120)	277	29.6
	High (> 0.120)	310	33.2
EUR	Low (< 0.710)	316	33.8
	Intermediate (0.710 - 0.870)	315	33.7
	High (> 0.870)	303	32.5
ASN	Low (< 0.030)	445	47.6
	Intermediate (0.030 - 0.050)	205	21.9
	High (> 0.050)	284	30.5
AME	Low (< 0.030)	404	43.3
	Intermediate (0.030 - 0.060)	231	24.7
	High (> 0.060)	299	32.0

Supplementary Table 2. Chemotherapy regimens.

Variable	Categories	n	%
Chemotherapy regimens	Quasar	103	30.0
	Mayo	114	33.2
	Folfox or Xelox	96	28.0
	Other (Flox)	30	8.7

Supplementary Table 3. Clinical Stage vs Chemotherapy.

Clinical Stage	Adjuvant chemotherapy		Total
	No<br>n (%)	Yes<br>n (%)	
0 / I	118 (94.4)	7 (5.6)	125 (100.0)
II	188 (50.8)	182 (49.2)	370 (100.0)
III	71 (22.0)	251 (78.0)	326 (100.0)
IV	164 (98.8)	2 (1.2)	166 (100.0)

Supplementary Table 4. Clinical Stage vs Chemotherapy regimens

Clinical Stage	Chemotherapy regimens				Total
	Quasar n (%)	Mayo n (%)	Folfox ou Xelox n (%)	Other (Flox) n (%)	
0 / I	1 (33.3)	2 (66.7)	0 (0.0)	0 (0.0)	3 (100.0)
II	71 (53.4)	59 (44.4)	2 (1.5)	1 (0.8)	133 (100.0
III	31 (15.0)	53 (25.7)	93 (45.1)	29 (14.1)	206 (100.0
IV	0 (0.0)	0 (0.0)	1 (100.0)	0 (0.0)	1 (100.0
